# Management of Symptomatic Venous Aneurysm

**DOI:** 10.1100/2012/386478

**Published:** 2012-04-01

**Authors:** Roberto Gabrielli, Maria Sofia Rosati, Andrea Siani, Luigi Irace

**Affiliations:** ^1^Department of Vascular Surgery, Policlinico Umberto I, Sapienza University of Rome, 00161 Rome, Italy; ^2^Department of Oncology, Policlinico Umberto I, Sapienza University of Rome, 00161, Italy

## Abstract

Venous aneurysms (VAs) have been described in quite of all the major veins. They represent uncommon events but often life-threatening because of pulmonary or paradoxical embolism. We describe our twelve patients' series with acute pulmonary emboli due to venous aneurysm thrombosis. Our experience underlines the importance of a multilevel case-by-case approach and the immediate venous lower limbs duplex scan evaluation in pulmonary embolism events. Our data confirm that anticoagulant alone is not effective in preventing pulmonary embolism. We believe that all the VAs of the deep venous system of the extremities should be treated with surgery as well as symptomatic superficial venous aneurysm. A simple excision can significantly improve symptoms and prevent pulmonary embolism.

## 1. Introduction

Isolated venous aneurysm (VA) is a focal dilation that communicates with normal vein through a single channel, and it should not be contained within a varicose segment. VAs have been reported in the intra- and extracranial veins, in the extremities, in the superior vena cava, and in the spleno-portal and common iliac system.

Venous aneurysms are usually uncommon. To our knowledge, the first characterization of this entity was from autopsy by Osler in 1915, while the first symptomatic popliteal VA with pulmonary embolism was described by Dahl et al. [1] in 1976.

Usually asymptomatic, VA can be detected when local lower extremity symptoms or embolic pulmonary episodes are present [2]. In those asymptomatic patients, the diagnosis is eventually performed for exclusion. The VAs of the extremities can be classified in two types: aneurysms of the deep and of the superficial venous systems. Venous aneurysm can be defined as a persistent isolated dilatation of twice the normal vein diameter [3] or three times in its normal size [4]. However, this definition does not cover all the VAs because many normal veins contain dilated segments that meet this definition (i.e., tibial and popliteal veins).

The natural history of the venous aneurysm remains poorly defined. Upper extremities VAs are usually asymptomatic and are most frequently treated for aesthetic reasons, while deep venous lower extremities aneurysms may be associated with thromboembolism and then surgery should be the recommended approach.

We report our 12-case retrospective analysis of acute pulmonary emboli due to VA's thrombosis and we underline the importance of accurate diagnosis and surgical repair in preventing further embolic events.

## 2. Materials and Methods

Between January 2004 and July 2010 we evaluated 46 patients with venous aneurysms of extremities. Aneurysms were located in the lower extremities (30 patients) and the upper extremities (6 patients) and, in the remaining 10 cases, were centrally localized (iliac vein, azygos, anonimous). Sixty eight percent of the primary lower extremities aneurysms occurred in the deep system.

From this cohort, 12 patients with primary symptomatic VA of the extremities and concomitant pulmonary embolism were identified (Table 1). Secondary superficial VA with varicose vein, venous malformation, arteriovenous malformation, and asymptomatic deep and superficial VA were excluded.

We analyzed the clinical features of these 12 patients, including sex, age, duration between the onset and the time of the diagnosis, the anatomical location, and the accompanying subjective symptoms. All the patients presented with signs and symptoms of pulmonary embolism and were examined by computed tomography angiographic (CTA) scan for pulmonary embolism, color duplex scan of extremities, and thrombophilic screening.

## 3. Results

**Figure 1:**
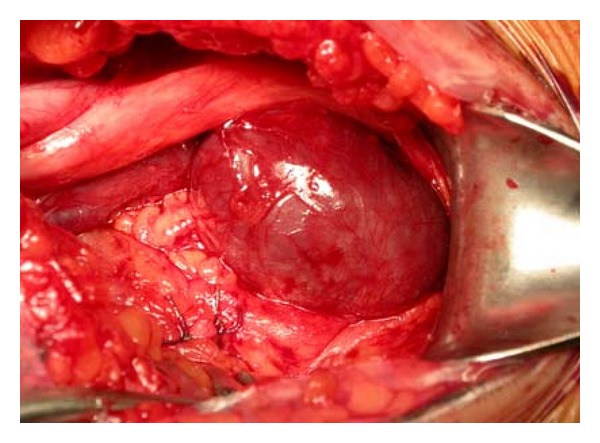
Intraoperative image shows a big popliteal vein aneurysm.

**Figure 2:**
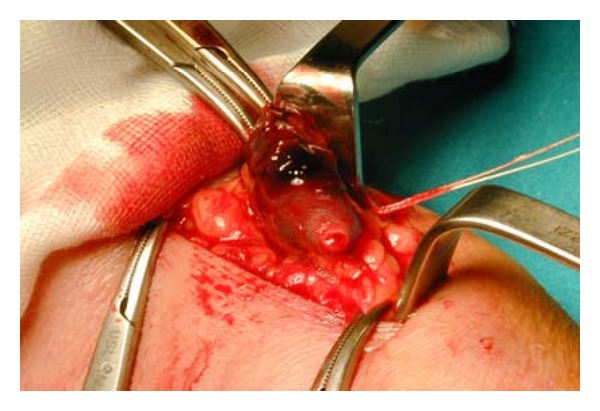
Intraoperative image shows the excision of cephalic vein aneurysm.

**Figure 3:**
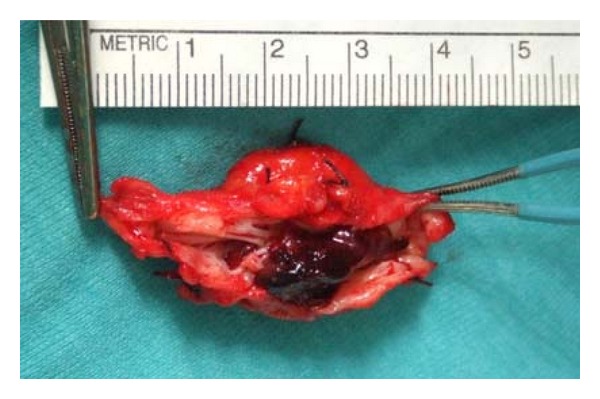
Small Saphenous vein aneurysm excised.

**Figure 4:**
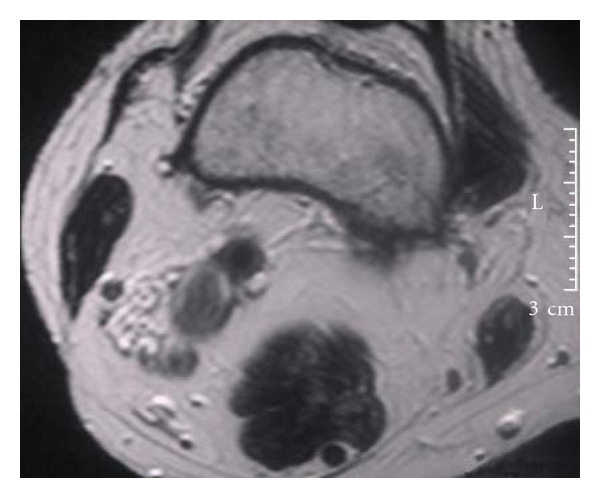
MR scan shows the small Saphenous vein aneurysm.

**Figure 5:**
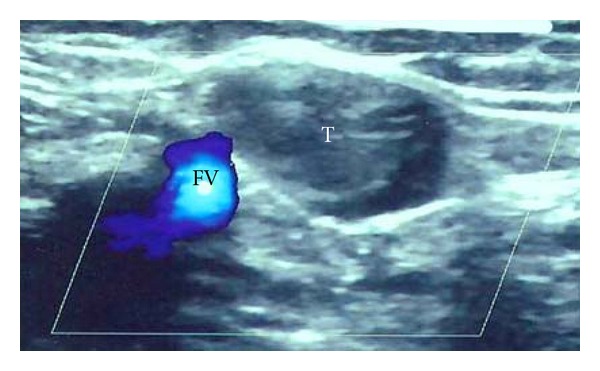
The US scan shows the great Saphenous vein aneurysm in communication with the femoral vein.

Venous aneurysm location was as follows: 6 cases in popliteal vein (Figure 1), 1 in posterior tibial vein, 2 cases in the great Saphenous vein (Figure 5), 2 cases in the cephalic vein (Figure 2), and small saphenous vein in the last case (Figures 3 and 4). Average aneurysm size was 3.9 cm ranging from 2,2 to 5,3 cm. Of the 12 patients included, a number of 5 man and 7 women were included with a median age of 38.9, ranging from 18 to 53 years. A temporary inferior cava vein (ICV) filter was placed to prevent the risk of embolism during aneurysm repair in those deep vein aneurysms.

In our series, the venous aneurysms were managed through a tangential excision or graft interposition or total excision.

**Figure 6:**
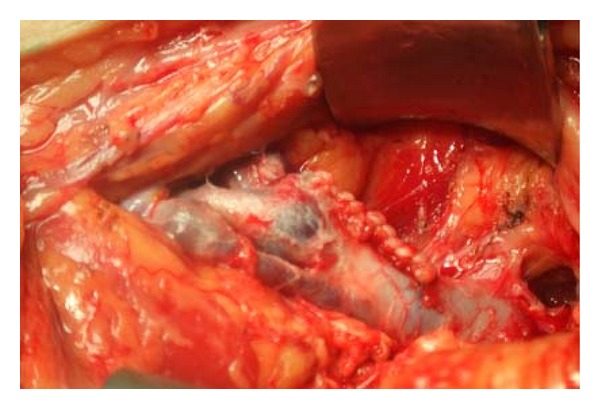
Intraoperative image showing tangential aneurysmectomy and lateral venorrhaphy.

Tangential excision was performed in four popliteal aneurysms (Figure 6) and in the tibial vein aneurysm while resection with interposition of autologous vein graft was performed in two popliteal cases. Total excision was performed in the remaining cases of superficial vein system aneurysm.

All the patients had an uneventful recovery and they were discharged with Acenocoumarol therapy for 3-4 months. In seven cases of deep vein aneurysms, a temporary ICV filter was placed and successfully removed after 2-3 months in five cases while this was not possible in the two remaining cases.

To a median follow-up of 18 months, a venous Doppler US demonstrated superficial and deep venous system patency without venous reflux in all the cases.

## 4. Discussion

Venous aneurysms have been reported in all major veins and they are often misdiagnosed as soft tissue masses or inguinal hernias. A soft tissue limb mass with change in size or Valsalva maneuver suggests a venous aneurysm of the extremity. The deep venous system localization appears to be more frequently associated with thromboembolism and worst venous morbidity than superficial system one.

The superficial venous system aneurysms incidence is described around at 0.1% [5], while the prevalence is up to 1,5% in 2000 patients from a single Vascular surgery Centre database [6].

The pathogenesis of the VAs is unknown; several mechanisms have been proposed ranging from reflux and venous hypertension, inflammation, infection, congenital vein wall weakness, mechanical trauma, and hemodynamic changes to localized degenerative change [7]. The most accepted theory is the focal normal connective tissue components loss of the vein wall. This could be due to a congenital underdevelopment or to a degenerative connective tissue loss with age [8].

This would end into wall weakness increasing the risk of dilatation. The endophlebohypertrophy and endophlebosclerosis are the main histologic feature of these processes [8]. Our findings adhere to those reported by other investigators [9, 10].

Moreover, a recent report examining venous aneurysm tissue suggested that the focal structural changes of the venous wall may be related to increased expression of select matrix metalloproteinases [11]. Our experience supports a local etiology process of the VAs with structural changes confined to the venous segment in which the aneurysm has formed.

These findings include a single irregular lumen, diminished smooth muscle component, increased fibrous tissue, fragmented elastin fibers, and few inflammatory cells infiltration.

Data from literature describe the incidence of venous aneurysms with concomitant pulmonary embolism at 24%–32% and chronic venous disease associated with VAs at the 76% [12]. Occasionally, superficial venous aneurysm could be associated with thromboembolism, but the real estimation is unknown; in fact two cases only were previously reported [6, 13]. Venous aneurysm rupture is a very rare complication [9].

Diagnosis is usually confirmed by duplex scan and followed by a CT scan, which allows the most correct assessment of VA.

Venous duplex imaging is the method of choice for diagnosis and it easily allows to evaluate venous aneurysms of the extremities and to define the size and the morphology of the aneurysm. However, we believe that before surgical repair, a CT scan is mandatory to investigate the deep venous system assessment and to define the venous anatomy [14].

Pulmonary embolic events represent the most frequent onset of venous aneurysm. The associated risk remains unpredictable and it may be unrelated to the presence or absence of thrombus on imaging. Our experience and a review from literature suggest that anticoagulation therapy may be ineffective in preventing pulmonary embolism [15].

The most common complications in venous aneurysms are deep venous thrombosis, thrombophlebitis, and recurrent pulmonary embolism; unfortunately, the diameter or the aneurysm shape cannot be considered solid parameter to predict these complications. Multiple episodes of pulmonary embolism in patient with a small saccular aneurysm have been in fact reported [16]. Our experience, in accordance with the literature, suggests that small deep venous aneurysms and large superficial venous system one can also be at risk.

Pulmonary emboli with severe hemodynamic instability may require thrombolytic therapy to improve cardiopulmonary function and to reduce thrombus burden in the deep venous system before the aneurysm has repair [17].

Preventive IVC filter placement can reduce the risk of embolism during deep vein aneurysm surgical repair [2, 18] or when thrombosis recurred in venous surgical area. Even though recurrent pulmonary embolism after surgery has never been reported yet, one case of fatal pulmonary emboli three hours later with a large femoral arteriovenous fistula excision has been described [19]. IVC filter can also be a valid option in elderly unfit patients who are not candidate to receive oral anticoagulation or in cases of severe PE with hemodynamic instability.

Aneurysmectomy and lateral venorrhaphy are a valid option to treat saccular venous aneurysms, while they can be resected occasionally only; in selected patients, a graft can be placed. Fusiform aneurysms can be treated with resection and end-to-end anastomosis or interposition graft and bypass or ligation of the proximal and distal vein. Superficial vein aneurysms can be treated by ligation of the afferent and efferent veins. Current endovenous ablation techniques are usually not feasible, owing to the aneurysm size and location [5]. Thus, treatment is primarily surgical and can be accomplished with simple ligation and excision [6].

After surgical repair, we recommend therapeutic anticoagulation for at least 3 months [20, 21]. Although the long-term results of surgery are yet unknown [22], data from literature on the primary patency rates are satisfactory, with no reports of recurrent pulmonary embolism following surgical repair. In one case only VA recurrence after lateral tangential aneurysmectomy has been previously reported [23].

Surgical repair has to be preferred in most of the patients with symptomatic (pain, severe edema, and thromboembolism) superficial or deep venous aneurysm and it can even be recommended in asymptomatic patients with saccular deep vein aneurysms (any size) and large fusiform aneurysms to prevent further thromboembolic events. Small and asymptomatic superficial venous aneurysms can be monitored by periodic Doppler ultrasounds.

## Figures and Tables

**Table 1:** Patient demographic and characteristic data.

	Sex	Age	Site	Medical history	D-dimer	Symptoms	Absolute size, cm	Histology	Intervention
Case 1	F	32	great saphenous vein	none	0.83 *μ*g/mL	increasing respiratory distress and left thoracic pain	3.1 × 2.9	aneurysm venous wall with endothelial denudation, attenuation of the elastic lamellae and medial fibrosis in areas of thrombus adherence	Ligation/excision

Case 2	M	42	popliteal vein	circulating phospholipid antibodies (aPL)	0.94 *μ*g/mL	acute shortness of breath	3.2 × 3.6	aneurysm venous wall characteristics with focal reduplication of the internal elastic lamina	tangential aneurysmectomy and lateral venorrhaphy

Case 3	M	43	popliteal vein	hypertension	1.21 *μ*g/mL	acute shortness of breath not associated with pleuritic chest pain or hemoptysis	2.9 × 3.6	aneurysm venous wall with thickened, fibrotic, moderately cellular intima adjacent to a densely fibrotic adventitia and rare smooth muscle	resection of venous aneurysm with interposition autologous vein graft

Case 4	M	19	cephalic vein	none	0.42 *μ*g/mL	increasing respiratory distress and right thoracic pain	2.9 × 3.5	Aneurysm wall with fragmentation, and attenuation of the elastic lamellae, loss of smooth muscle cells,	Ligation/excision

Case 5	M	46	small saphenous vein	Leg varicose vein, CAD	0.63 *μ*g/mL	acute shortness of breath	2.6 × 2.3	characteristics of varix	Ligation/excision

Case 6	M	42	posterior tibial vein	none	0.69 *μ*g/mL	acute shortness of breath	3.2 × 2.6	attenuation of the elastic lamellae, loss of smooth muscle cells	tangential aneurysmectomy and lateral venorrhaphy

Case 7	M	42	popliteal vein	circulating phospholipid antibodies (aPL)	0.89 *μ*g/mL	acute shortness of breath	3.2 × 3.6	aneurysm venous wall characteristics with focal reduplication of the internal elastic lamina	tangential aneurysmectomy and lateral venorrhaphy

Case 8	M	43	popliteal vein	hypertension	1.05 *μ*g/mL	acute shortness of breath not associated with pleuritic chest pain or hemoptysis	2.9 × 3.6	aneurysm venous wall with thickened, fibrotic, moderately cellular intima adjacent to a densely fibrotic adventitia and rare smooth muscle	resection of venous aneurysm with interposition autologous vein graft

Case 9	M	19	cephalic vein	none	0.57 *μ*g/mL	acute shortness of breath	2.9 × 3.5	Aneurysm wall with fragmentation, and attenuation of the elastic lamellae, loss of smooth muscle cells,	Ligation/excision

Case 10	M	46	small saphenous vein	Leg varicose vein, CAD	0.71 *μ*g/mL	increasing respiratory distress and right thoracic pain	2.6 × 2.3	characteristics of varix	Ligation/excision

Case 11	M	42	popliteal vein	circulating phospholipid antibodies (aPL)	0.82 *μ*g/mL	acute shortness of breath	3.2 × 3.6	aneurysm venous wall characteristics with focal reduplication of the internal elastic lamina	tangential aneurysmectomy and lateral venorrhaphy

Case 12	M	43	popliteal vein	hypertension	1.32 *μ*g/mL	acute shortness of breath not associated with pleuritic chest pain or hemoptysis	2.9 × 3.6	aneurysm venous wall with thickened, fibrotic, moderately cellular intima adjacent to a densely fibrotic adventitia and rare smooth muscle	resection of venous aneurysm with interposition autologous vein graft

CAD: Coronary artery disease.
